# Dual-theoretical framework integrating the stress process model and family systems theory-based psychological intervention for caregivers of children and adolescents with mood disorders: study protocol for a randomized controlled trial

**DOI:** 10.3389/fpubh.2026.1920774

**Published:** 2026-10-13

**Authors:** Wei Lin, Hongyan Wu, Xuan Liu, Yangyang Yu, Xiaoqing Ye, Lin Lin

**Affiliations:** 1 School of Nursing, Guangdong Medical University, Dongguan, China; 2 Affiliated Hospital of Guangdong Medical University, Zhanjiang, China

**Keywords:** caregivers, children and adolescents, mood disorders, protocol, psychological intervention, randomized controlled trial

## Abstract

**Background:**

Globally, the prevalence of mood disorders among children and adolescents is rising, with the associated burden affecting patients, families, and society. Primary caregivers—mostly parents—experience sustained psychological stress and their mental health directly influences patients’ treatment adherence and clinical outcomes. Despite this, research on interventions has predominantly focused on the caregivers of children with autism spectrum disorder or attention-deficit/hyperactivity disorder. The caregivers of youth with mood disorders, particularly bipolar disorder, have been largely neglected. Moreover, theory-driven intervention programs for this population remain scarce.

**Methods:**

Grounded in the stress process model and family systems theory, we developed a comprehensive psychological health intervention for caregivers of children and adolescents with mood disorders and designed a randomized controlled trial to evaluate its effectiveness. A total of 216 caregivers will be recruited and randomly assigned at a 1:1 ratio to either the intervention group receiving the theory-based program or the control group receiving routine psychological nursing and health education. Outcomes include anxiety, depression, parenting stress, coping style, family functioning, and self-efficacy. These will be assessed at baseline, immediately post-intervention, and at 1-month follow-up. Semi-structured interviews will be conducted within 2 weeks post-intervention to capture caregivers’ lived experiences.

**Discussion:**

Adhering to the SPIRIT guidelines, this protocol targets primary caregivers of children and adolescents with mood disorders, a critically important yet under-recognized population. Based on evidence synthesis, five caregiver needs identified in the scoping review have been consolidated into four theory-based intervention targets within the SPM–FST framework: cognitive adjustment and belief restructuring, emotion regulation and stress coping, parent–child communication and family functioning enhancement, and group mutual support and social support. The intervention will be delivered via face-to-face group sessions, and its effectiveness will be evaluated using a mixed-methods approach combining quantitative scales and qualitative interviews. If shown to be effective and feasible, this program may provide empirical evidence for developing multi-level family caregiver support systems.

**Clinical trial registration:**

https://www.chictr.org.cn/, identifier ChiCTR2600123504.

## 1 Introduction

Mood disorders, including depressive disorders and bipolar disorder, in children and adolescents have become a major public health concern worldwide, and the number of affected young people has increased continuously in recent years (1). These disorders tend to start early in life, last for a long time, and return at a high rate after improvement (2–5). One systematic review of data from 29 countries found that among children and adolescents, rates of depressive disorders at mild-to-moderate, moderate-to-severe, and severe levels were 21.3, 18.9, and 3.7%, respectively. This means that more than one out of every five children and adolescents globally have experienced depressive episodes or had depressive symptoms at some point (6). Another systematic review covering studies from seven countries found that the rate of bipolar disorder was 1.8% (7). In China, a national survey in 2023 among schoolchildren aged 6–16 years found that 3.2% of participants had mood disorders, and adolescents had a higher rate of these disorders than younger children (8).

Evidence shows that when a child or teenager experiences a mood disorder, adverse effects can appear at individual, family, and societal levels. At the individual level, mood disorders can negatively affect how children control their emotions, how they do in school, and how they get along with other people. These problems may carry over into their adult years, harming their mental health and making it harder for these individuals to fit into society (3, 8–11). Additionally, mood disorders are often associated with a greater likelihood of developing physical health problems, such as heart disease and metabolic syndrome. They are also linked to the development of other mental health problems, such as anxiety disorders and drug or alcohol problems. These associations all lead to mood disorders having a particularly heavy total burden of illness. At their worst, these disorders can result in suicidal thoughts or behaviors, with some data showing that approximately 15% of people with major depressive disorder or bipolar disorder end up dying by suicide (12). At the family level, the burden of raising a child or adolescent with a mood disorder increases substantially, accompanied by a greater financial burden. Individuals caring for the child or adolescent often exhibit signs of depression and anxiety at high rates (13). At the level of society, the weight of mood disorders is mostly seen in the cost associated with these disorders. More than one trillion US dollars are lost globally each year due to major depressive disorder alone; in the United States, the annual loss due to bipolar disorder is more than 45 billion US dollars (12).

Most caregivers of children and adolescents with mood disorders are the parents, making family-based care the predominant care model for these young patients. Daily tasks that fall on caregivers include ensuring the child/adolescent takes medication as prescribed, watching for changes in symptoms, providing feedback to health professionals about treatment progression, and offering emotional support. Caregivers must also deal with ongoing relapses, behavior troubles, and poor social skills in the patient, all of which can simultaneously amount to an enormous degree of stress and strain on parents or caregivers. This can then negatively impact their mental health. One study across multiple countries found that family members of adolescents with major depressive disorder had a greater likelihood of developing depression themselves, having mood swings, and abusing alcohol (14). Another study showed that, when compared with caregivers of children without mood disorders, caregivers for children with bipolar disorder experienced greater stress from raising their child, had worsening depression symptoms over time, and exhibited more antisocial behaviors (15).

In China, findings similar to those mentioned above have been reported. The Survey Report on Pain Points in the *Treatment and Rehabilitation of Depression in Children and Adolescents*, published in 2024, states that 33.8% of the parents of adolescents with depression showed signs of depression themselves, and among these parents, 20.2% were at a high risk of developing depression (16). Gao et al. (17) and Yang et al. (18) reported anxiety symptoms in 34.8 and 37.6% of caregivers of adolescents with depression, depression symptoms in 41.8 and 42.7%, and body-related complaints in 38.4 and 42.4%, respectively. Wang et al. conducted a study of 198 family caregivers of patients with bipolar affective disorder. The authors found an overall burden across all measures that was above the medium level among these caregivers, and their resilience scores were much lower than those in the general adult population of China (19). Li et al. reported very similar results (13).

Family caregivers are central to the treatment and rehabilitation of children and adolescents with mood disorders, playing a critical role in patients’ clinical progression and improvement in quality of life. First, in terms of treatment adherence, children and adolescents often do not fully comprehend their illness, and because their cognitive abilities are still developing, they do not have sufficient motivation to maintain long-term treatment. Therefore, caregivers become responsible for ensuring the treatment plan is correctly followed over the long term. Second, from the perspectives of improved mental health and social life, the home environment most strongly shapes how children and adolescents develop emotionally. How caregivers feel, how they cope with problems, and how parents and children interact and relate all have a direct effect on whether the patient feels safe and wishes to improve. On top of this, family systems theory posits that when children and adolescents have mental disorders, these problems can be understood by examining how family members interact and how the family functions as a whole. Thus, interventions or programs focused only on the child or adolescent usually do not bring about the best results, which is why family-centered approaches to treating and helping patients improve are crucial (20).

Although mental health disorders in children and adolescents have received increased attention worldwide, studies until now have mostly focused on the young patients themselves. Insufficient attention has been paid to the psychological distress experienced by the caregivers of these youth, or how this pain might interfere with improvement in the patient. To date, research among caregivers of children and adolescents with mental disorders has been largely aimed at those with autism spectrum disorder (ASD) (21–30) or attention-deficit/hyperactivity disorder (ADHD) (31–40). This emphasis may have arisen because these are long-lasting conditions that place a heavy burden on caregivers. ASD and ADHD often require urgent intervention, and these conditions can adversely affect family functioning at a deep level (41). In terms of format, psychological interventions for the parents of children and adolescents with ASD/ADHD are primarily delivered face-to-face. These interventions typically include cognitive behavioral therapy, parent behavioral training, acceptance and commitment therapy, and mindfulness-based interventions.

In contrast to the above studies, those involving caregivers of children and adolescents with mood disorders remain notably scarce (42–47). A handful of studies among parents of children with depressive disorders have sought to address the emotional state of parents using approaches such as Balint groups, Satir family therapy, and family-centered talk therapy. However, these studies had small sample sizes, interventions were at the exploratory stage, and a clear and well-tested research plan was lacking. Moreover, almost no past studies exist on mental health programs for caregivers of children and adolescents with bipolar disorder, highlighting a large gap in this research area.

Herein, a mixed-methods framework integrating quantitative and qualitative methods will be used to assess a psychological support program for the caregivers of children and adolescents affected by mood disorders. The primary outcome measure is anxiety, and secondary outcomes comprise depression, parenting stress, coping style, family functioning, and self-efficacy. Additionally, qualitative data will be gathered via semi-structured interviews to explore the impact of the intervention on caregivers, as well as their perceptions and suggestions regarding the program. It is hypothesized that, following the intervention, caregivers will exhibit reduced levels of anxiety and depressive symptoms, decreased parenting stress, a shift toward more positive coping style, and improvements in family functioning and self-efficacy.

## 2 Methods and analysis

### 2.1 Study design and randomization

This study is designed as a randomized controlled trial (RCT), comprising an intervention group and a control group. The intervention group will receive the comprehensive psychological health intervention program developed for this study, and the control group will receive routine psychological nursing care combined with health education. The intervention group will receive the intervention for 2 months and will participate in eight structured sessions. Outcome assessments are scheduled at three time points: T0 (baseline, pre-intervention), T1 (immediately post-intervention), and T2 (at 1-month follow-up). The control group will be assessed at the same three time points. An overview of the study procedures is provided in Figure 1, and detailed information regarding the study design is provided in Table 1.

**Figure 1:**
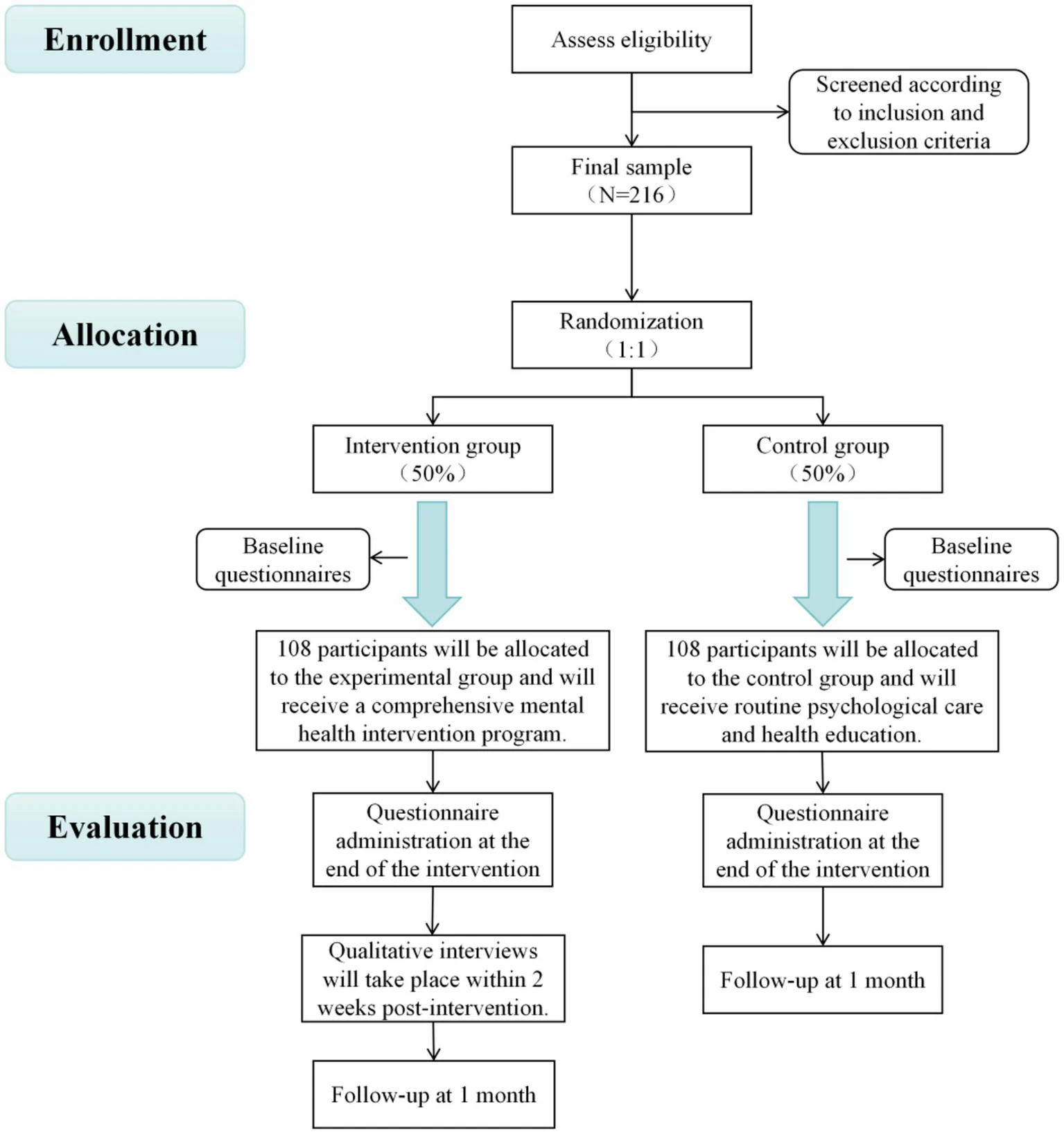
Trial design.

**Table 1:** Timeline for study enrollment, intervention, and evaluation.

Item	Study period					
	Recruitment	Allocation	Intervention		Follow-up	
Timepoint	−T_1_	0	Month 0	Month 2	Within 2 weeks after the intervention	Month 3
Enrollment						
Informed consent	√					
Eligibility screening	√					
Allocation		√				
Intervention						
Intervention group			⬌			
Control group			⬌			
Assessments						
Qualitative interviews					√	
Anxiety			√	√		√
Depression			√	√		√
Parenting stress			√	√		√
Coping style			√	√		√
Family functioning			√	√		√
Self-efficacy			√	√		√

### 2.2 Ethics and study participants

The ethics committee of the Clinical Trial Service Center of the Affiliated Hospital of Guangdong Medical University has approved this study protocol. All study participants will be the primary caregiver of a patient at this hospital. Participant recruitment began in May 2026 and is expected to be completed by 1 August 2026. If a potential participant meets the inclusion criteria and is willing to take part in the study, they can be enrolled after receiving a thorough explanation of the study in a face-to-face setting and signing an informed consent form. In this study, “caregiver” is defined as an “informal caregiver,” following the definition used by the World Health Organization. Under this definition, an informal caregiver refers to an immediate family member who provides unpaid care. In this study, caregivers are immediate family members who provide unpaid care to children and adolescents with mood disorders (48).

#### 2.2.1 Inclusion criteria

The following inclusion criteria shall be applied in this study. (1) Having a child or adolescent (aged ≤ 19 years) under the participant’s care who has received a definitive diagnosis of a depressive disorder or bipolar and related disorders from an attending physician, based on the Diagnostic and Statistical Manual of Mental Disorders, Fifth Edition, Text Revision (DSM-5-TR) (49). All diagnoses in this study will be made by physicians in the Department of Psychiatry and Psychology of the Affiliated Hospital of Guangdong Medical University. The diagnosing physicians will all hold a professional title of attending physician or above and will have been engaged in clinical psychiatric work for more than 5 years. Before the start of the study, all diagnosing physicians will receive standardized training in use of the DSM-5-TR. (2) The caregiver is an immediate family member of the patient or a designated primary guardian responsible for the main caregiving duties. (3) The caregiver has adequate reading comprehension ability and can communicate effectively. (4) The caregiver voluntarily agrees to participate in the study and has signed an informed consent form.

#### 2.2.2 Exclusion criteria

Caregivers meeting any of the following criteria will be excluded from this study. (1) Those with cognitive or communication disorders. Specifically, caregivers with moderate or greater intellectual disability, moderate-to-severe dementia, or cognitive impairment will be excluded. This will be confirmed by clinical diagnosis or investigator assessment. In addition, caregivers judged by the investigator as likely to affect the caregiver’s understanding of the study procedures or the informed consent form will be excluded from the study. Finally, caregivers with severe communication disorders (e.g., uncorrected deaf-mutism or aphasia) that preclude effective communication with the investigator in the absence of professional interpretation or auxiliary devices will also be excluded. (2) Recent participation in similar interventions, such as participation in any structured group intervention or systematic psychoeducational program targeting caregiver mental health within the 6 months prior to recruitment. (3) Current participation in other interventional studies, such as a psychosocial intervention or drug clinical trial. (4) Other circumstances deemed by the investigator to render the caregiver inappropriate for participation.

#### 2.2.3 Criteria for intervention discontinuation

If any of the following circumstances occur, discontinuation of the participant’s intervention will be determined by the principal investigator: (1) missing two consecutive sessions, or a total of three sessions, and failing to complete the required make-up sessions; or (2) discontinuation decided by the investigator based on safety or scientific considerations (e.g., serious adverse events, serious protocol violations). Participants who discontinue the intervention will not be regarded as withdrawing from the study; these participants will continue to complete the follow-up assessments at each time point as originally planned, and their data will be included in the intention-to-treat (ITT) analysis.

#### 2.2.4 Withdrawal criteria

If any of the following circumstances occur, the participant will be regarded as having completely withdrawn from the study: (1) voluntary withdrawal of informed consent by the participant; or (2) loss to follow-up, defined as being unable to reach the participant after at least three contact attempts using each of three different methods (e.g., telephone, text message, and email) over a 2-week period, without any explicit refusal to continue participation. In this case, all data collection will be terminated, the existing data will be included in the ITT analysis according to the original group assignment, and missing data will be handled according to the prespecified strategies in the statistical analysis plan.

### 2.3 Sample size

#### 2.3.1 Determination of the effect size

The primary analysis will use a linear mixed-effects model (LMM; see Statistical methods section). For sample size estimation, an independent-samples *t*-test was used as a conservative approximation, as the LMM is expected to have greater power due to repeated measures, within-subject correlation, and baseline adjustment. With G*Power 3.1, a two-sided *α* = 0.05, power of 1 − *β* = 0.80, and an allocation ratio of 1:1, anxiety was the primary outcome for sample size estimation. The effect size was determined by integrating the evidence from two meta-analyses. Tsang et al. (50) conducted a meta-analysis among caregivers of children with anxiety or depression and reported a pooled effect on anxiety with Hedges’ *g* = −0.26. Sheng et al. (51) conducted a meta-analysis among caregivers of children with chronic diseases and reported a pooled effect on anxiety with standardized mean difference = −0.49. The former had a study population highly consistent with that of the present study, but the interventions lacked a clear theoretical basis and showed high heterogeneity, which resulted in a conservative effect estimate. Although the latter study focused on caregivers of children with chronic diseases, it shares clinical similarity with the present study in the core feature of the “caregiving burden.” Both effect sizes fall within the small-to-moderate range. Given that the present study protocol is rigorously grounded in the stress process model (SPM) and family systems theory (FST), with predefined intervention targets, the expected effect should be greater than that reported by Tsang et al. Therefore, d = 0.49 was taken as the expected effect size, which remains within the small-to-moderate effect-size range and does not overestimate the expected treatment effect.

#### 2.3.2 Conservative selection of the clustering parameter

Considering the clustering effect of the group-based intervention, and referring to an intraclass correlation coefficient (ICC) range of 0.02–0.10 reported by Kivlighan et al. (52), combined with the highly structured and manualized characteristics of the present protocol, a conservative ICC of 0.04 was adopted; the design effect was calculated based on the minimum group size of m = 8: Deff = 1 + (8 − 1) × 0.04 = 1.28.

#### 2.3.3 Adjusted sample size

Based on the above parameters, the minimum required sample size at the individual level was 134 participants. After adjustment for clustering (×1.28), the required sample size was 172 participants. Considering an estimated dropout rate of 20% and rounding to a whole number, the final target sample size was set at 216 participants (108 per group).

### 2.4 Recruitment

Participants will be recruited for the study via three main channels. (1) Online and offline recruitment ads will run at the same time. Online ads will be publicized through the official WeChat public account of the hospital’s psychiatry department and through WeChat support groups set up for caregivers. Flyers and posters will be posted in locations where caregivers tend to gather, such as outpatient waiting areas and inpatient wards. The ads will describe the study and the intervention, who is eligible for participation, the study duration, possible benefits, and how to contact the research team. (2) Face-to-face recruitment. Psychiatric specialist nurses on the research team will identify potentially eligible caregivers during regular ward rounds and outpatient clinical practice. Nurses will provide caregivers with a short introduction to the study and invite them to learn more so they can decide whether they wish to enroll in the study. (3) Recommendations from health professionals. Physicians caring for patients in the psychiatry department and nurses responsible for these patients’ day-to-day care will identify caregivers who meet the inclusion criteria and who, in their judgment, may benefit from additional psychological support.

Caregivers will be informed that their participation in the study is completely voluntary. Once a caregiver gives their written informed consent, they will be randomly allocated to one of the two study arms. If the target of 216 participants has not been reached by the end of the planned recruitment window, recruitment will continue until the required sample size is achieved. The trial was prospectively registered with the Chinese Clinical Trial Registry (ChiCTR2600123504) on April 27, 2026. Participant enrolment commenced only after registration, in May 2026.

### 2.5 Intervention

#### 2.5.1 Intervention group: comprehensive psychological health intervention program

The intervention program in this study is an original theory-driven project, with SPM as the core theoretical framework, integrated with FST. SPM conceptualizes the stress process as comprising four domains—stressors, mediators, stress reactions, and stress outcomes (53–54)—and has been widely applied to explain stress mechanisms and guide intervention development (55–57). FST complements this framework by emphasizing that families function as interactive systems, suggesting that psychological interventions should target family communication, emotional expression, and relational balance to restore adaptive family functioning.

Guided by these two theoretical frameworks, and informed by our preliminary needs assessment and literature review, we operationalized the intervention as follows: care activities were identified as the primary stressor; caregiver anxiety and depression were specified as the stress outcomes; and emotion regulation and stress coping, cognitive adjustment and belief restructuring, group mutual support and social support, and parent–child communication and family functioning enhancement were designated as the four mediating targets for intervention. These four mediators were directly translated into core intervention components, with specific session content designed to modify each target mechanism. The theoretical framework is illustrated in Figure 2, which identifies the core stress-process pathways through which the intervention is hypothesized to exert its effects.

**Figure 2:**
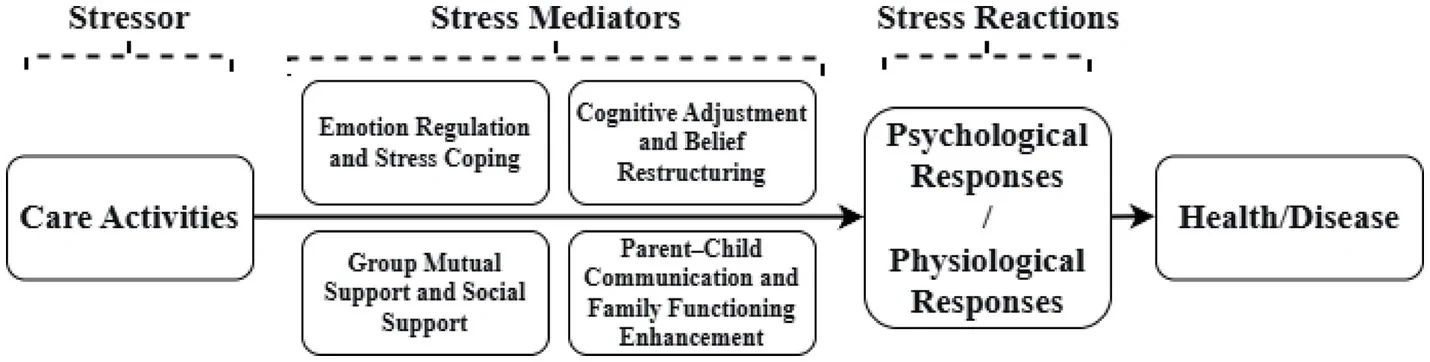
Theoretical framework integrating the stress process model and family systems theory. Care activities are specified as the primary stressor. The pathway from stressor to stress reactions is mediated by four modifiable targets: emotion regulation and stress coping, cognitive adjustment and belief restructuring, group mutual support and social support, and parent–child communication and family functioning enhancement. These mediators are directly targeted by the intervention. Outcomes include psychological responses (e.g., anxiety or depression), physiological responses, and health/disease status. Arrows indicate the hypothesized causal pathways through which the intervention is expected to exert its effects.

The intervention will be conducted in the psychological counseling room of the Department of Psychiatry and Psychology at the Affiliated Hospital of Guangdong Medical University. The intervention will last for 2 months, with a total of eight weekly sessions, each comprising 90–100 min of face-to-face group psychological intervention. The specific intervention content includes psychoeducation, cognitive-behavioral skills training, mindfulness-based stress reduction practices, and family-systems-oriented parent–child communication training. The detailed content of the intervention, with explicit specification of the theoretical target and expected mechanism for each session (see also Table 2 for a session-by-session mapping).

**Table 2:** Session-by-session mapping of intervention components to theoretical constructs and expected outcomes.

Week	Session theme	Active components	Theoretical construct(s) targeted	Expected outcome(s)
1	Establishment of a support team	5-min mindful breathing, warm-up/icebreaking activities, group introduction, group contract establishment, and “Caregivers’ Voice Circle” activity (sharing caregiving experiences, fostering mutual empathy, and enhancing group cohesion)	Group mutual support and social support	Proximal outcomes: parenting stress Distal outcomes: anxiety and depression
2	Disease-related knowledge acquisition	5-min mindful breathing, health education on knowledge related to child and adolescent mood disorders, followed by a Q&A session; “Myth vs. Fact” interactive activity (clearly contrasting misconceptions and correct knowledge to reduce stigma and guilt)	Cognitive adjustment and belief restructuring	Proximal outcomes: parenting stress, self-efficacy Distal outcomes: anxiety and depression
3	Emotion recognition and self-regulation	5-min mindful breathing, emotion recognition and labeling; “emotion body mapping” activity; skills training (diaphragmatic breathing and progressive muscle relaxation)	Emotion regulation and stress coping	Proximal outcomes: parenting stress, coping style, self-efficacy Distal outcomes: anxiety and depression
4	Cognitive restructuring and belief modification	5-min mindful breathing, introduction of the ABC^a^ theory to illustrate the importance of cognition; presentation of irrational automatic thoughts and their common manifestations; “Caregivers’ Beliefs Workshop” (the ABCDE^b^ model of REBT^c^ applied to real-life caregiving scenarios as practical exercises)	Cognitive adjustment and belief restructuring	Proximal outcomes: parenting stress, coping style, self-efficacy Distal outcomes: anxiety and depression
5	Stress coping and behavioral skills training	5-min mindful breathing, stress assessment and personal stress mapping activity; explanation of the four principles of caregiver stress coping (30 min of personal time, boundary setting, help-seeking behavior, and three rapid stress-relief techniques)	Emotion regulation and stress coping	Proximal outcomes: parenting stress, coping style, self-efficacy Distal outcomes: anxiety and depression
6	Parent–child communication and improvement of family functioning	5-min mindful breathing, introduction to communication strategies (“four-step communication model” and “universal communication sentence templates”); one-to-one mindfulness-based listening practice; role-playing of high-frequency conflict scenarios	Parent–child communication and family functioning enhancement	Proximal outcomes: parenting stress, coping style, self-efficacy, family functioning Distal outcomes: anxiety and depression
7	Gentle acceptance and resilience rebuilding	5-min mindful breathing, introduction to the concept of acceptance; reading an “acceptance declaration”; writing an acceptance letter to oneself based on a structured template	Emotion regulation and stress coping	Proximal outcomes: parenting stress, coping style, self-efficacy Distal outcomes: anxiety and depression
8	Consolidation of gains and ongoing support	5-min mindful breathing, review of all previous sessions and guided questioning; “Harvest Tree” reflection activity; skills consolidation (portable coping toolkit); introduction of follow-up support and evaluation plan; closing activity designed to generate emotional warmth and serve as a structured termination of the program	Group mutual support and social support	Proximal outcomes: parenting stress Distal outcomes: anxiety and depression

^a^ABC refers to emotional ABC theory (Activating event–Belief–Consequence). ^b^ABCDE refers to the five steps: Activating event, Belief, Consequence, Disputation, Effect. ^c^REBT refers to Rational Emotive Behavior Therapy.

The intervention implementation team will comprise the principal investigator, one head nurse from the Department of Psychiatry and Psychology, and two psychiatric specialty nurses. The head nurse of the psychiatry and psychology department will serve as the lead facilitator. This team member is a psychology specialty nurse who has completed systematic professional training in psychological intervention methods, including cognitive behavioral therapy, mindfulness-based stress reduction, and family systems therapy, and who possesses the clinical qualifications to integrate multiple intervention techniques. The remaining team members will assist with on-site facilitation, intervention procedure implementation, data management, and other related work.

This intervention is designed only for caregivers of children and adolescents with mood disorders and does not affect any treatment received by children who require hospitalization or are currently hospitalized. If child or adolescent is discharged, the caregiver can continue to complete the entire study. The study plan is to recruit 216 participants, with 108 participants each in the intervention group and control group. The 108 participants in the intervention group will be allocated to 12 groups, with approximately 8–10 participants per group. The specific number of member of group will be flexibly adjusted according to the recruitment pace to ensure that each batch size remains within an appropriate range.

#### 2.5.2 Control group: routine psychological nursing care and health education

Participants in the control group will receive routine psychological nursing care and health education provided by psychiatric specialist nurses in the ward, conducted concurrently with the intervention group. The care for the control group will not follow a fixed frequency or a structured intervention framework; rather, it will reflect the standard care practice currently delivered in a clinical setting. The main components of this care are as follows: (1) Emotional support and companionship. Nurses will provide a supportive environment in which caregivers can share their feelings and concerns, with an emphasis on active listening, empathy, and companionship. (2) Basic health education. During routine ward rounds and nursing care, nurses will offer verbal health education to caregivers as needed. The content will cover fundamental knowledge about mood disorders, including causes, symptoms, treatment, medication adherence, and general caregiving guidance. (3) Educational materials. Printed health education brochures related to mood disorders and caregiver self-care will be made available in the ward for caregivers to access independently. After completion of the study, caregivers in the control group who express interest will be given the option to participate in the comprehensive psychological intervention program.

### 2.6 Blinding and randomization

This study will use an assessor-blinded (single-blind) design. Outcome assessors and statisticians will be blinded to group allocation and will remain blinded throughout data collection and the primary analysis. However, due to the nature of the intervention, the blinding of participants and intervention providers is not feasible. To minimize bias, outcome assessors will conduct independent evaluations without access to group allocation information.

For randomization, an independent researcher not involved in the study will generate a random sequence using IBM SPSS 27.0. Sequentially numbered, opaque, sealed envelopes will be prepared to conceal group assignments. When a new participant is enrolled, recruitment staff will open the next envelope in sequence, and the participant will be allocated to either the intervention or control group based on its contents. A total of 216 participants will be randomly divided between the two groups at a 1:1 ratio.

### 2.7 Outcomes

Outcome data will be collected from both participant groups at three assessment points: T0 (baseline, pre-intervention), T1 (immediately post-intervention), and T2 (at 1-month follow-up). Within 2 weeks of intervention completion, a subset of participants from the intervention group will also take part in semi-structured interviews. The program will be assessed along two dimensions: feasibility and effectiveness. Feasibility metrics cover recruitment rate, retention, dropout rate, and adherence. Effectiveness endpoints comprise anxiety, depression, parenting stress, coping style, family functioning, and self-efficacy.

#### 2.7.1 Primary outcome

Anxiety will be assessed using the Zung Self-Rating Anxiety Scale (SAS). The scale has 20 items with responses given on a four-point Likert scale (1 = “none or a little of the time” to 4 = “most or all of the time”). The total score ranges from 20 to 80. Cut-off scores are used to determine the level of anxiety, with higher scores indicating worse anxiety: 50–59 indicates mild anxiety, 60–69 moderate anxiety, and 70 or above severe anxiety (58). In a study among caregivers of patients with lung cancer, the SAS showed a Cronbach’s *α* of 0.93 (59).

#### 2.7.2 Secondary outcomes

Depression will be measured using the Zung Self-Rating Depression Scale (SDS). Much like the SAS, the SDS includes 20 items, with items rated on a 4-point Likert scale; the total score ranges from 20 to 80. For the SDS, cut-offs marking the severity of depression are as follows. Scores of 53–62 indicate mild depression, 63–72 moderate depression, and 73 or above severe depression; the higher the score, the more severe the depressive symptoms (60). The version of the SDS that has been adapted for use in China has shown satisfactory validity and reliability (61). In a sample of caregivers of patients with depression, Li et al. reported a Cronbach’s *α* of 0.87 for the SDS (62).

Parenting stress will be captured using the Parenting Stress Index–Short Form (PSI-SF). This is a 36-item tool covering parental distress, parent–child dysfunctional interaction, and difficult child, with 12 items in each dimension. Items are rated on a 5-point scale, from 1 = “strongly disagree” to 5 = “strongly agree.” Scores in each dimension range from 12 to 60, and the total score across all 36 items ranges from 36 to 180. Higher scores indicate greater parenting stress felt in that dimension (63). The Chinese version of the PSI-SF is reported to have sound psychometric properties; the Cronbach’s *α* for the full scale is above 0.80 (64–66), making this a suitable instrument for assessing stress among caregivers.

Caregivers’ coping style will be determined using the Simplified Coping Style Questionnaire. The scale has 20 items across two dimensions: 12 items focus on positive coping and 8 on negative coping. For each item, respondents are asked how often they turn to a particular way of coping when experiencing difficulties or setbacks. Responses are given on a 4-point Likert scale, with 0 meaning “never used” and 3 “often used” (67). The Chinese version of this scale has favorable reliability and validity (68–69). The overall Cronbach’s *α* is 0.90, with 0.89 for the positive coping subscale and 0.78 for the negative coping subscale (70).

Family functioning will be examined using the Family APGAR (Adaptation, Partnership, Growth, Affection, and Resolve) Index, which has five dimensions. Each dimension is measured using just one item, with responses given using a 3-point Likert scale: 0 = “hardly ever,” 1 = “sometimes,” and 2 = “often.” The total score across all five items is from 0 to 10. Scores of 7–10 suggest good family functioning, 4–6 moderate dysfunction, and scores of 0–3 signal serious dysfunction (71). Lyu et al. used this scale among patients with cerebrovascular disease and found that it had good validity and test–retest reliability, with Kappa values above 0.7; the scale was satisfactory for every dimension except Adaptation, as was the total scale (72).

Self-efficacy will be measured using the General Self-Efficacy Scale. This is a straightforward, one-dimension scale with 10 items. Each item is rated using a 4-point Likert scale, from 1 = “not at all true” to 4 = “completely true.” The total score ranges from 10 to 40, with higher scores indicating greater self-efficacy (73). This scale has been used among primary caregivers of patients with cancer by Luan et al., who reported a Cronbach’s *α* of 0.87 (74).

### 2.8 Data handling and analysis plan

#### 2.8.1 Data collection

This study will adopt a data collection method combining quantitative and qualitative approaches. Quantitative outcomes will be measured using structured questionnaires. Each scale selected for use in this study is a standardized tool that has undergone validity and reliability testing. Before formal data collection, the research team will review a standard set of instructions with each participant regarding how to complete the questionnaires to ensure the data are accurate and consistent. Within 2 weeks after intervention completion, a subset of participants from the intervention group will be selected to take part in semi-structured qualitative interviews. This selection will be conducted using purposive sampling combined with maximum variation sampling. These interviews are designed to explore in depth participants’ experience and acceptance of the intervention, as well as their perceived benefits. Based on the sample size requirements of descriptive qualitative research and the principle of data saturation, the planned sample size is 15–20 participants. An interview guide will be developed based on the research objectives and relevant literature and will be used in a round of pilot interviews. These interviews will be conducted by members of the research team who have research backgrounds in nursing and psychology, have received systematic training in qualitative research, and who are familiar with the intervention program in this study but are not involved in implementation of the intervention.

#### 2.8.2 Statistical methods

Quantitative data analysis will be performed using IBM SPSS 27.0 and R software (lme4 package). The primary outcome is the change in SAS scores from baseline to each follow-up time point; secondary outcomes include changes in SDS and PSI-SF scores, coping style scores, family APGAR index scores, and self-efficacy scores.

Descriptive statistics and between-group comparisons will be conducted. Continuous variables will be examined for normality using the Shapiro–Wilk test. Normally distributed variables will be described using mean ± standard deviation, and between-group comparisons will be conducted using independent-samples *t*-tests. Non-normally distributed variables will be described using median (interquartile range), and between-group comparisons will be carried out using the Mann–Whitney *U* test. Categorical variables will be described as frequency (percentage), and between-group comparisons will be made using the *χ*^2^ or Fisher’s exact test.

In the primary efficacy analysis, longitudinal efficacy will be analyzed using a linear mixed model (LMM), with parameters estimated by restricted maximum likelihood. The fixed effects will include group, time (T0/T1/T2), group × time interaction, and the baseline value of each outcome. A random intercept at the individual level will capture within-participant correlation. Because the intervention is delivered in small groups, we will apply cluster-robust standard errors with small group as the clustering variable to adjust for within-group non-independence, rather than including a small-group random intercept, as the number of groups is limited and the small group is the unit of intervention delivery rather than the unit of randomization. The group × time interaction is the key analytic parameter; if it is significant, simple effect analyses will be further conducted. The normality and homogeneity of variance of the model residuals will be assessed using diagnostic plots. For between-group comparisons, Cohen’s d and its 95% confidence interval will be reported.

Secondary outcomes will be analyzed as exploratory analyses, interpreted in conjunction with effect sizes and confidence intervals, without correction for multiplicity. All hypothesis tests will be two-sided, with *p* < 0.05 considered statistically significant.

Regarding the analysis populations, primary analyses will follow the ITT principle, and all randomized participants with at least one post-baseline assessment will be included. Discontinuation of the intervention will not warrant exclusion. A per-protocol analysis will be conducted as a sensitivity analysis.

In terms of handling missing data, before the formal analysis, the proportion of missing data at each time point and the reasons for dropout will be described. The baseline characteristics of participants who completed all follow-up assessments will then be compared with those of participants who dropped out. The primary analysis will be done using the LMM, which can use all available observations for parameter estimation; if the dropout rate is >15%, a repeated analysis based on multiple imputation will be performed as a sensitivity analysis.

For qualitative data analysis, audio recordings from the semi-structured interviews with caregivers will be transcribed verbatim; anything in the transcripts that could identify any participant will be removed prior to the analysis. The transcripts will then be analyzed following the six-phase thematic analysis framework put forward by Braun and Clarke in 2006 (75): (1) repeatedly reading the text to become familiar with the raw data; (2) generating initial codes; (3) searching for themes; (4) reviewing themes; (5) defining and naming themes; and (6) writing and interpreting the qualitative analysis results. NVivo will be used during the analysis to assist with data organization and management, coding, and the retrieval of relevant materials.

To integrate quantitative and qualitative results, this study will adopt an explanatory sequential design, with the quantitative approach as the primary strand and the qualitative approach as the secondary strand. The qualitative interviews will be conducted within 2 weeks after the intervention, aiming to explain and deepen the main findings of the quantitative study. Integration will be achieved through a joint display, in which the quantitative statistical results and qualitative themes will be presented side by side. Cross-validation and complementary interpretation will be carried out to clarify whether the intervention is effective and why it is either effective or ineffective.

### 2.9 Quality control

#### 2.9.1 Data monitoring

This study will be conducted at a single center, will pose low risk, and will not involve any drugs; it is purely a psychological intervention. The program delivers psychological support and health education, with no invasive or high-risk medical procedures. Thus, no major safety issues affecting study participants are expected. Given the low-risk nature of this study, a formal, independent data monitoring committee will not be established. Instead, the research team itself will be responsible for study implementation, data management, and participant safety, strictly in keeping with ethics requirements.

#### 2.9.2 Intervention quality control

Before the start of the study, all personnel implementing the intervention will have completed a 3-day standardized training program led by the principal investigator and head nurse of the Department of Psychiatry and Psychology. The training covers the study protocol and operational procedure manual, core intervention skills, on-site facilitation, data management, and standardized adverse event reporting procedures. During the study, the team will hold a supervision meeting once weekly, chaired by the principal investigator, to review specific details of intervention implementation.

To ensure intervention fidelity, the following measures will be adopted: (1) a standardized intervention manual will be developed, specifying the objectives, content, facilitation instructions, and time allocation of each intervention session; (2) all intervention personnel are required to receive unified training and follow the manual during implementation; (3) fidelity will be assessed by an independent research assistant who is not involved in intervention delivery, using a structured fidelity checklist through on-site observation of each session; and (4) implementation records will be completed promptly after each intervention session, and the quality of implementation will be continuously reviewed in combination with retrospective reflection.

To systematically track the non-standardized care exposure received by the control group, a specialty nurse is required to complete a “Routine Care Contact Record Form” after each care contact with caregivers in the control group, recording the date, duration, format, and main topics of discussion. Control group participants will complete a “Caregiver Support Resource Utilization Log” every 2 weeks, self-reporting the types and frequency of various sources of support received. After the study, the care contact records will be retrospectively verified using the medical record system.

To control between-group contamination, the intervention will be delivered by trained research team members in a separate area, with timing staggered from routine ward activities; the control group will receive the usual care from ward nurses, with no overlap in providers. Intervention-specific materials will be labeled “for research use only” and kept outside public areas, whereas the control group will receive a general information booklet without the core therapeutic components. Participants will be instructed to not share intervention content with others in the informed consent form. To monitor potential contamination, contact records for the control group will include an item on whether participants have been exposed to the intervention materials or content.

To control provider contact differences, communication with the control group will be standardized in both content and duration, and proactive in-depth psychological intervention is prohibited. All contacts are to be fully documented. Contact duration will be compared descriptively between groups to confirm comparability, but it will not be adjusted for in the primary ITT analysis to avoid overadjustment bias.

#### 2.9.3 Qualitative research quality control

To control the influence of researchers’ backgrounds on data collection and interpretation, each interviewer will complete an entry in a reflexive journal within 24 h after each interview. Before the data analysis begins, the researchers will state in writing their own position in relation to the topic of study. During data analysis, continuous self-reflection shall be maintained. Specifically, the potential influence of the researchers’ own views and interactions with the participants on data interpretation will be recorded and examined to reduce subjective bias.

Two researchers will code the data independently and the degree of coding consistency will be reported. Any disagreements will be adjudicated through discussion by the research team until consensus is reached.

The full-process analytical memos—from the original transcripts through initial coding, category generation, and final theme development—shall be retained, ensuring that the analysis process is open and transparent and that the results are traceable and can be verified.

### 2.10 Potential risks

Although this study involves no invasive procedures or direct physical risk to participants, several potential issues associated with this type of research study are worth mentioning.

The study involves both psychological intervention and psychological assessment. During the interviews or group sessions, participants may find themselves discussing or recalling difficult experiences, the burden of caregiving, and how these make them feel. This could lead to feelings of sadness, anxiety, embarrassment, mood swings, or other types of psychological discomfort.

The study delves into caregivers’ personal experiences and topics involving family life. The research team will follow strict protocols regarding maintenance of confidentiality, and all data will be handled using coded identification numbers to avoid exposing participants’ identities. Even so, participation in group-based sessions or data storage and transmission involve a small risk of information disclosure.

Participation in this study involves attending weekly sessions over a period of 2 months, which could place a burden of time and inconvenience on participants’ personal schedules and daily routines.

## 3 Discussion

Most past research on psychological health interventions for caregivers of children and adolescents with psychiatric disorders has focused on the caregivers of young people with neurodevelopmental conditions, particularly ASD and ADHD. Those caring for children and adolescents with mood disorders have been infrequently investigated. The handful of studies involving this group have tended to include small samples, use exploratory intervention models, and lack standardized protocols (43–48). The number of children and adolescents with mood disorders continues to rise, with effects at the individual, family, and societal levels. Against this backdrop, the primary caregivers of these patients—most often the parents—experience ongoing high levels of psychological stress, which adversely affect their own mental health. This in turn directly influences patient treatment and recovery. Yet few studies have focused on the caregivers of children and adolescents with mood disorders, especially bipolar disorder. With social support for these caregivers being very limited, the present study aims to broaden research into this group, in line with the growing shift toward family-centered, systems-based approaches to mental health intervention. Our objective is to provide a scientific and evidence-based basis for the development of more family-oriented caregiver support systems.

In this study, we aim to develop and evaluate a comprehensive psychological health intervention program for caregivers of children and adolescents with mood disorders that draws on two theoretical anchors—the SPM and FST. The SPM concentrates at the individual level, mapping the origin of stressors, mediators of their impact, and how stressors feed into psychological health outcomes. Use of this model can provide clear targets within the intervention design. The FST emphasizes that psychological stress does not occur in a vacuum but is woven into the patterns of family dynamics. Thus, intervention must go beyond the individual to encompass the family environment and family relationships. Combining these two theoretical bases can extend their reach to new areas of mental health practice by offering a novel framework that can facilitate understanding and support caregivers in a more comprehensive, multi-layered fashion.

An early version of the intervention program was developed based on preliminary investigations, a literature search, and a scoping review, and was then revised through Delphi expert consultation. To determine whether the intervention is feasible and effective, an RCT is planned among caregivers of children and adolescents with mood disorders. The program will be delivered face-to-face in a group psychological counseling format, shaped to fit the real-world needs of caregivers. The scoping review identified five key caregiver needs: knowledge about mood disorders, cognitive appraisal, coping style, family functioning, and social support. These five needs were consolidated into four theory-based intervention targets within the integrated SPM–FST framework: (1) knowledge about mood disorders and cognitive appraisal were merged into cognitive adjustment and belief restructuring; (2) coping style was addressed through emotion regulation and stress coping; (3) family functioning was targeted through parent–child communication and family functioning enhancement; and (4) social support was targeted through group mutual support and social support. This mapping ensures that each intervention component is explicitly linked to a defined theoretical mechanism. The intervention aims to ameliorate anxiety, depression, and stress among caregivers and to improve their coping, self-efficacy, and family functioning. The control group will receive routine psychological nursing care and health education as part of standard clinical practice, enabling comparison between the theory-driven intervention and standard care. After the intervention, qualitative interviews will be conducted alongside quantitative assessments to capture caregivers’ experiences and feedback. This will help identify facilitators and barriers to broader implementation and factors that enhance adherence, thereby maximizing program effectiveness.

### 3.1 Limitations

Several limitations should be acknowledged. First, the use of convenience sampling may introduce selection bias, as caregivers who volunteer to participate may differ from those who decline in terms of motivation, help-seeking behavior, or baseline psychological distress. In addition, this study was conducted at a single center within a specific cultural and healthcare context, which may limit the generalizability of our findings to other regions or healthcare systems with different family structures, caregiving norms, or mental health service delivery models. Second, all effectiveness outcomes are self-reported by caregivers, which may introduce reporting bias and common method variance, the results should therefore be interpreted with caution. Third, due to the nature of the group-based intervention, only outcome assessors will be blinded, which may introduce performance and detection bias. To minimize this, assessors will not deliver the intervention themselves, participants will be instructed not to disclose their allocation before assessments, and qualitative interviews will only be conducted after all quantitative assessments are completed. Fourth, the multi-component nature of the intervention, while theoretically grounded, prevents us from isolating the specific contribution of each element. This is a challenge inherent in complex intervention research. Fifth, the 1-month follow-up is a relatively short period for evaluating the long-term sustainability of effects. For outcomes such as family functioning, coping style, and self-efficacy, which may require longer periods to stabilize, this follow-up duration may be insufficient to determine whether intervention effects are sustained. Sixth, the control condition comprises routine care with basic emotional support and health education. These components may provide non-specific benefits that attenuate between-group differences, thereby yielding conservative effect estimates. Future studies should consider extended follow-up periods (e.g., 6 or 12 months), multi-center designs, factorial approaches to identify active ingredients, and attention-matched controls to enhance condition contrast and isolate specific intervention effects.
